# Polysaccharides from the root of *Angelica sinensis *promotes hematopoiesis and thrombopoiesis through the PI3K/AKT pathway

**DOI:** 10.1186/1472-6882-10-79

**Published:** 2010-12-21

**Authors:** Chang Liu, Jianqin Li, Fan Yi Meng, Simon X Liang, Ruixia Deng, Chi Kong Li, NH Pong, Ching Po Lau, Sau Wan Cheng, Jie Yu Ye, Jian L Chen, ST Yang, Haixia Yan, Shilin Chen, Beng H Chong, Mo Yang

**Affiliations:** 1 Department of Hematology, Nanfang Hospital, Southern Medical University, Guangzhou, PR China; 2 Institute of Medicinal Plant Development, Chinese Academy of Medical Science, 151 MaLianWa North Road, Beijing, 100193, PR China; 3 LKS Faculty of Medicine, The University of Hong Kong, Hong Kong, PR China; 4 The First Affiliated Hospital, Sun Yat-Sen University, Guangzhou, PR China; 5 Center for Vascular Research, Department of Hematology, St George Hospital, University of New South Wales, Sydney, Australia; 6 The Chinese University of Hong Kong, Prince of Wales Hospital, Hong Kong, PR China

## Abstract

**Background:**

Dozens of Traditional Chinese Medicine (TCM) formulas have been used for promotion of "blood production" for centuries, and we are interested in developing novel thrombopoietic medicines from these TCMs. Our previous studies have demonstrated the hematopoietic effects of DangGui BuXue Tong (DBT), a formula composed of *Radix Angelicae Sinensis *and *Radix Astragali *in animal and cellular models. As a step further to identify and characterize the active chemical components of DBT, we tested the hematopoietic and particularly, thrombopoietic effects of polysaccharide-enriched fractions from the root of *Radix Angelicae Sinensis *(APS) in this study.

**Methods:**

A myelosuppression mouse model was treated with APS (10 mg/kg/day). Peripheral blood cells from APS, thrombopoietin and vehicle-treated samples were then counted at different time-points. Using the colony-forming unit (CFU) assays, we determined the effects of APS on the proliferation and differentiation of hematopoietic stem/progenitor cells and megakaryocytic lineages. Using a megakaryocytic cell line M-07e as model, we analyzed the cellular apoptosis progression with and without APS treatment by Annexin V, Mitochondrial Membrane Potential and Caspase 3 assays. Last, the anti-apoptotic effect of APS on cells treated with Ly294002, a Phosphatidylinositol 3-Kinse inhibitor (PI3K) was also tested.

**Results:**

In animal models, APS significantly enhanced not only the recovery of platelets, other blood cells and their progenitor cells, but also the formation of Colony Forming Unit (CFU). In M-07e cells, we observed the anti-apoptotic effect of APS. Treatment by Ly294002 alone increased the percentage of cells undergoing apoptosis. However, addition of APS to Ly294002-treated cells significantly reduced the percentage of cells undergoing apoptosis.

**Conclusions:**

APS promotes hematopoiesis and thrombopoiesis in the mouse model. This effect likely resulted from the anti-apoptosis activity of APS and is likely to involve the PI3K/AKT pathway.

## Background

Thrombocytopenia (an abnormal decrease in the number of platelets in circulatory blood) is frequently developed in hematological and cancer patients who undergo bone marrow suppression or infiltration resulting from chemotherapy or radiotherapy. This condition may lead to haemorrhage and fatality [1]. In severe cases, platelet transfusion may be required to prevent or stop bleeding. However, platelet transfusion may induce the formation of anti-platelet antibodies, and the transmission of both viral and bacterial infection. Until today, no effective treatments for thrombocytopenia are clinically available. Our long term goal is to identify novel thrombopoietic agents from Traditional Chinese Medicine (TCM) formulations or products for further development.

Dozens of TCM formulations have been used for promotion of "blood production" for centuries and have shown favorable effects on thrombocytopenia [2,3]. Previously, we specifically characterized Danggui Buxue Tong (DBT) and demonstrated that it has significant effects on promoting thrombopoiesis [4]. Many chemical components have been identified in DBT. Among them is the polysaccharide fraction of *Angelica sinensis *(APS). The structure of the APS-iron complex (APIC) had been proposed to be a polynuclear ferrihydrite core chelated firmly by an encircling framework of APS chains, forming a core molecule, which is surrounded by a removable outer protective sheath of colloidal APS. The molecular formula of APIC was proposed to be {[(Fe_2_O_3_·2.2H_2_O)1043(APS)32](APS)12}, with MW = 270000 Da [5]. Recent pharmacological studies demonstrated that APS had radio-protective effects in irradiated mice through modulation of proliferating response of hematopoietic stem cells [2]. In gastrointestinal system, APS was known to be protective against ethanol- or indomethacin-induced mucosal damage [6]. It was also reported that *Angelica sinensis *crude extract increased the proliferation of gastric epithelial cells through modulation of several proliferation-related genes, including EGF, ODC, and c-Myc [7-9]. In cancer cells, APS has been reported to possess anti-tumor effects [10,11].

Although several studies have demonstrated the general hematopoietic activity of APS, no study has been performed to specifically determine its thrombopoietic effects. In addition, the mechanism of action of APS's effect has not been investigated. Here, we tested if APS can promote thrombopoiesis. Firstly, we confirmed that APS can promote the recovery of various hematopoietic lineages in a mouse model. Secondly, we found that APS can promote the proliferation of megakaryocytic progenitor cells and bone marrow stromal cells *in vitro*, which might directly lead to the platelet production. Thirdly, APS can also inhibit the apoptosis of megakaryocytic cells *in vitro*. In separate studies, our lab has found that Phosphatidylinositol 3-kinase/AKT is likely to be involved in the APS's effects. As a result, a PI3 kinase inhibitor was used to treat the cells with or without APS. We found that APS reversed the effects of Ly294002, a PI3 kinase inhibitor. In summary, the current study has demonstrated the promoting effect of APS on thrombopoiesis and this effect might involve the PI3K/AKT pathways. The work from this study laid the foundation for the development of possible APS based therapeutic strategies.

## Methods

### Plant Materials and Chemical Reagents

The roots of *Angelica sinensis *(Oliv.) Diels, (RAS, Chinese name: Danggui) used in our experiments were purchased from Minxian County, Gansu Province, China. These samples are currently deposited in the Molecular Chinese Medicine Laboratory, University of Hong Kong as voucher # mcm-011. The standard RAS materials used for quality control was purchased from National Institute for the Control of Pharmaceutical and Biological Products, China (batch number: 120927-200411). Anthrone and glucose were purchased from Guoyao Enterprise group, Tianjing Damao Chemicals Company (Tianjing, China). Sulfuric acid was purchased from Sigma-Aldrich (St Louis, MO).

### Quantitative Analysis of Plant Materials

The experimental and standard RAS materials were dried at 60°C, ground and powdered by an electric mill (TR-02B, Rong Tsong, Taiwan) and sieved through a mesh (size 20). The powders were then ground with KBr (AR, Perkin Elmer, UK) and pressed into pellets using a KBr hand press (Specac, UK). The pellets were analyzed by a Spectrum 100 (PerkinElmer, UK) under the following conditions, wavelength range: 400 to 4000 cm^-1^; spectral resolution: 4 cm^-1^. Data analysis was performed using software PerkinElmer Spectrum (Version 6.2.0).

### Preparation and Quantitative Analysis of APS Samples

RAS polysaccharides (APS) were prepared according to the method described by Cho et al [6]. Briefly, 100 grams of RAS were extracted three times by boiling in water for four-hours each time. All water extracts were pooled and mixed with ethanol to a final concentration of 75% v/v to precipitate the polysaccharide-enriched fraction (APS). The total amounts of polysaccharides were measured using the anthrone-sulfuric acid assay. Briefly, the standard glucose solution was prepared in MiliQ water at serial concentrations of 0, 0.010, 0.020, 0.040, 0.060 and 0.080 mg/ml. Four ml of anthrone-sulfuric acid solutions (0.2 g/ml) were added to a 1 ml APS solution and each of the six standard solutions respectively. All solutions were kept and mixed on ice. The mixtures were boiled in a water bath for 15 min and left at room temperature for 10 min before they were analyzed using a UV/VIS Spectra Lambda 35 (PerkinElmer). Three replicate analyses were conducted.

### Testing Endotoxic Activity by Gel-clot Tachypleus Amebocyte Lysate (TAL) Test

All materials required for TAL test including Tachypleus Amebocyte Lysate, control standard endotoxin (10 EU/ml), TAL reagent water, test tubes and pipette tips free of detectable endotoxin were purchased from Zhanjiang Bokang Marine Biological Co., LTD (Guangdong, P.R.China). The gel-clot TAL tests were preformed according to the method described in Chinese Pharmacopoeia 2010 edition. Briefly, 100 μl series dilutions of control standard endotoxin or APS extracts were mixed with 100 μl TAL reagents. The mixed solutions were incubated at 37°C for 60 minutes. The formation of the gel was scored by turning each test tube upside down. If the gel remained a piece, it is considered a solid gel formation. Otherwise, it is considered a failed gel formation. Endotoxin is measured in Endotoxin Units per milliliter (EU/ml). One EU equals approximately 0.4 ng/ml of endotoxin solution in our experiment.

### Radiation-induced Haematocytopenic Model

Seven to eight week-old male Balb/c mice were obtained from Charles River Japan (Yokohama, Japan) and given free access to food and water. The treatment of animals were conducted following protocols provided by Hong Kong government and ethical permissions for the studies were granted by the Animal Research Welfare Committee, The Chinese University of Hong Kong. A haematocytopenia with thrombocytopenia model was established using 4-Gy-irradiated mice as described previously [12,13]. APS (2.5 mg/day) and TPO (0.25 μg/day) were given by injection (IP) daily for 21 days starting from the day after radiotherapy in these mice. Peripheral blood platelets, white blood cells (WBC) and red blood cells (RBC) from APS, TPO and water control groups were analyzed on days 0, 7, 14, and 21. On day 21, the bone marrow cells were harvested for colony-forming unit (CFU) assays. We measured the plasma TPO levels on day 21. Bone marrow samples were frozen in cryomolds and performed on 5-μm sections. The slides were stained with Giemsa staining. Twenty-five random high-power fields from each bone marrow sample were chosen and blindly quantified for histological examination.

### Murine Colony-Forming Unit (CFU) Assay

The assay was performed as described previously [14]. Colony-forming unit-granulocyte macrophage (CFU-GM), burst-forming unit/colony-forming unit-erythroid (BFU/CFU-E), and colony-forming unit-mixed (CFU-GEMM) were cultured in methylcellulose (1%) supplemented with fetal calf serum (FCS, 30%), 1% BSA, 0.1 mM β-mercaptoethanol, 3 IU/ml erythropoietin, 10 ng/ml granulocyte macrophage-colony stimulating factor, 10 ng/ml interleukin-3, and 50 ng/ml SCF. Murine bone marrow cells (2 × 10^5 ^cells/ml) were seeded in triplicate and incubated for 7 days. Colonies were scored blindly.

### Murine Colony-Forming Unit-Megakaryocytes (CFU-MK) Assay

Murine bone marrow cells (2 × 10^5 ^cells) were cultured using the plasma clot culture method [14,15]. The culture medium contains 1% deionized bovine serum albumin (BSA) (Sigma, Mo USA), 0.34 mg CaCl_2_, 10% citrated bovine plasma (Sigma), 100 μg penicillin, 50 μg streptomycin and IMDM with different concentrations of APS, TPO and IL-3 in a total volume of 1 ml. The cells were incubated at 37°C under 5% CO_2 _for 7 days and the number of CFU-MK derived colonies was counted using acetyl-choline esterase (AchE) staining method after 7 days. The colonies were further stained with haematoxylin to count the CFU-GM derived colonies. A CFU-MK colony was defined as a cluster of 3 or more AchE positive cells and a CFU-GM colony was considered as a cluster of 40 or more cells.

### Murine Bone Marrow Colony-Forming Unit-Fibroblast (CFU-F) assay

The assay was performed as described previously [16,17]. Briefly, mouse bone marrow cells (1 × 10^6 ^cells) were seeded in 2 ml of IMDM with 10% FCS in triplicates. Cultured cells were incubated at 37°C and 5% CO_2 _in a fully humidified atmosphere with or without APS for 9 days. Fibroblastoid colony-forming cells (CFU-F) assay were used to determine the number of bone marrow-derived fibroblastoid [16]. Briefly, adherent cells were stained with Giemsa staining. The number of CFU-F colonies was counted under a light microscope. An aggregate containing more than 10 fibroblasts were counted as a CFU-F colony. Effects of APS and other cytokines such as fibroblast growth factor (FGF, 50 ng/ml), platelet-derived growth factor (PDGF, 50 ng/ml), or vascular endothelial growth factor (VEGF, 50 ng/ml) were also examined using the CFU-F assay.

### Annexin V, Caspase 3, and Mitochondrial Membrane Potential Analyses of M-07e Cells by Flow Cytometry

The assays were performed as described previously [14]. Briefly, the megakaryoblastic cell line M-07e (American Type Culture Collection, Manassas, VA, http://www.atcc.org) was maintained in IMDM supplemented with GM-CSF (20 ng/ml) and 10% FCS. Apoptotic cell death was induced by cytokine and serum depletion. We added APS (200 μg/ml) to the cultures and then the cells were incubated for 72 hours. Apoptotic cell death was examined using the annexin V-FITC/PI, active Caspase 3-PE, and JC-1 ApoAlert reagent kits (BD Biosciences, San Diego, http://www.bdbiosciences.com) according to the manufacturer's instructions. Ten thousand events were acquired for each sample and analyzed by flow cytometry using the Lysis II software (FACScan; BD Pharmingen).

### Statistical Analysis

Treatment groups were compared using analysis of variance, paired *t *test or Wilcoxon signed rank test, depending on data distribution, using the JMP software (SAS, Cary, NC). A *p *value of <0.05 was considered statistically significant. All values were expressed as mean ± SEM. Statistical significances were denoted with three different symbols: "*" (*p <*0.05), "#" (*p <*0.01) and "+" (*p <*0.001).

## Results

The current experimental studies include three parts: (1) quality control of APS; (2) effects of APS on mice; (3) effects of APS on the apoptosis of cells. In part 2, mice (Normal) were subjected to radiotherapy to create a bone-marrow-damaged model (Control). Then we study the effects of APS on the recovery of various hematopoietic, especially those thrombopoietic cells. All treatments were conducted on these control mice. In part 3, apoptosis were induced in cells by serum depletion. The effects of APS were tested on these apoptotic cells. In our experiments, TPO was used as the positive control. And a PI3K inhibitor was used to test if APS's effects implicate the PI3K pathway.

### (1) Quality control of APS

**Figure 1:**
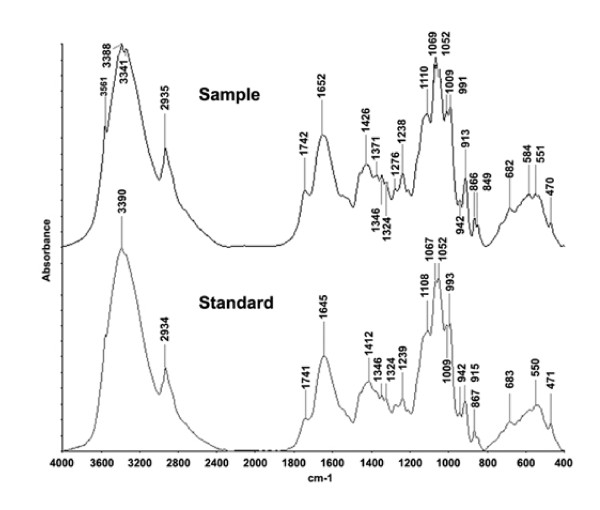
**Spectrums of a RAS sample and the RAS standard**. The wavelengths are shown on the x axis and the values of absorbance are shown on the Y axis. The wavelengths for the identified peaks are indicated.

Since the APS preparations usually contain various chemical compositions, qualitative and quantitative defining the experimental materials is important. This is to ensure that various preparations of RAS have consistent biological activities and any follow-up studies can reproduce the results generated in previous studies. Here, we used two methods to define our APS preparations. First, the RAS samples were subjected to UV spectral analysis and the spectrum were compared to that of a standard. The IR spectrums of our RAS experimental material and the RAS standard material are shown in Figure 1. The above analyses were repeated five times and the correlation coefficients of the spectrums were found to be greater than 0.9973 (data not shown). Then we measured the total amounts of polysaccharides in the APS preparations using the anthrone-sulfuric acid assay. Glucose was used as the standard and the standard curve was found to be y = 0.0069 * x + 0.0045 with a correlation coefficient (R2) of 0.9992. By comparing to the standard curve, polysaccharides were found to represent 90% of the total APS preparations.

Both bacterial endotoxins or lipopolysaccharide (LPS) and (1,3)-β-D-glucan can trigger TAL gelation, which is used for the detection and semi-quantification of the LPS or β-glucan content of the APS preparation. We found that the solid gel was formed for APS dilutions at concentrations of 5, 1.667, 0.556, and 0.185 mg/ml. In comparison, gelation occurred at concentrations of 0.125 and 0.06 EU/ml for the control standard endotoxin. So the LPS or β-glucan content of the APS preparation was estimated to be approximately 0.06/0.185 or 0.32 EU/mg of APS extracts. This is equivalent to 0.128 ng endotoxin in 1 mg of APS.

### (2) The effects of APS on hematopoiesis and thrombopoiesis in myelosuppressed mice

#### Effects of APS on blood cell counts

**Figure 2:**
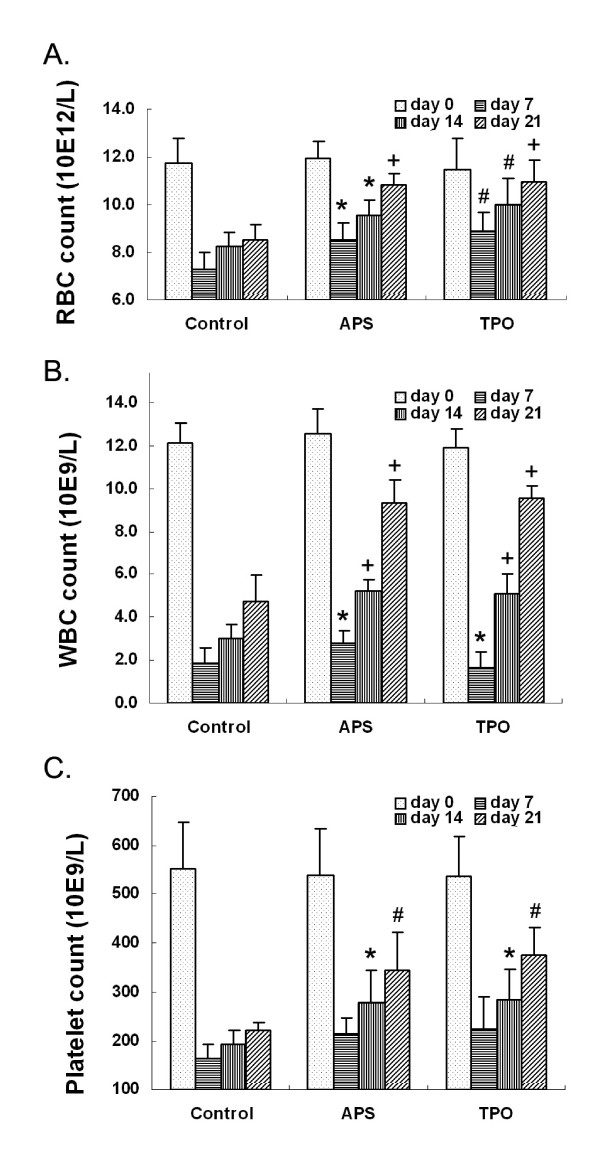
**Effects of APS on blood cell counts of irradiated mice model**. Mice (n = 6) were irradiated on day 0 and then treated with vehicle (Control), APS (10 mg/kg/day, APS) and TPO (1 ug/kg/day, TPO). The blood was taken and the numbers of (A). red blood cells (RBC), (B). white blood cells (WBC) and (C). platelets were counted. The statistical tests were carried out on the cell counts between the corresponding tests and the control groups. *: *p <*0.05; #: *p <*0.01 and +: *p <*0.001.

As shown in Figure 2A, RBC counts of APS treated mice showed significant increased recovery on days 7, 14 and 21. Similarly, RBC counts in TPO treated mice also showed significant increased recovery on days 7, 14 and 21, suggesting that APS and TPO have similar effects on the recovery of RBC cells. The changes of WBC cell counts in the experiment were shown in Figure 2B. WBC counts in APS treated mice showed significantly increased recovery on days 7, 14 and 21. Similarly, WBC counts in TPO treated mice also showed significantly increased recovery on day 7, 14 and 21, suggesting that APS and TPO have similar effects. Mice treated with APS at 10 mg/kg/day showed significantly higher platelet number on days 14 and 21 (Figure 2C) and accelerated the platelet recovery compared with that of the control mice. Similarly, TPO-treatment at 1 μg/kg/day significantly increased the platelet counts comparing to that of control on day 14 and 21. This thrombocytopoietic activity of APS was similar to that of TPO as there were no significant differences in the platelet counts between samples treated with APS and TPO.

#### Effects of APS on the total body weight and organ weight

**Figure 3:**
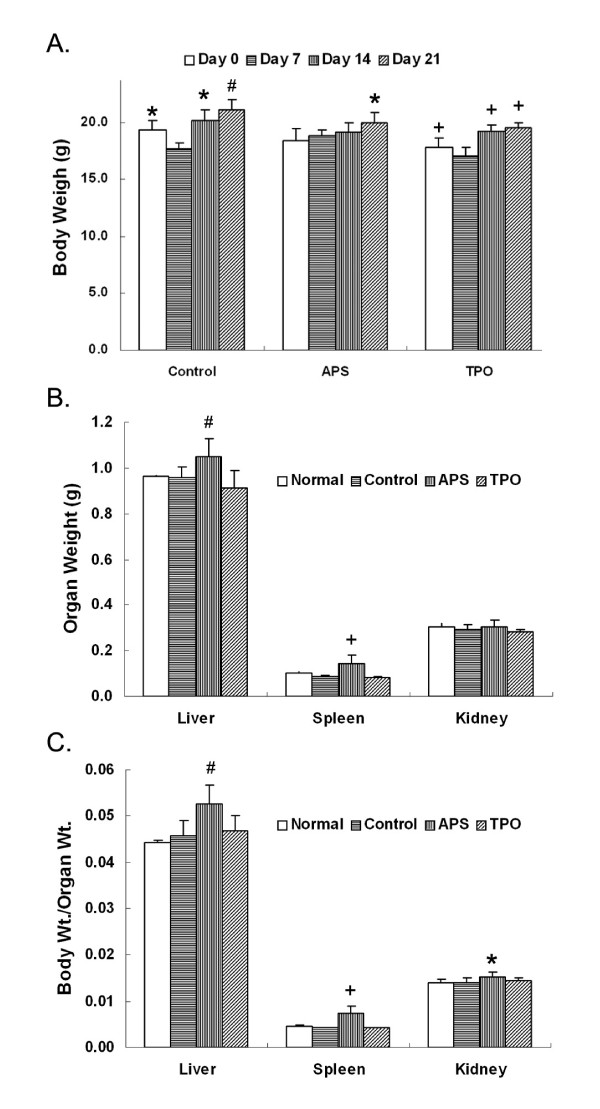
**Effects of APS on the animal body size and organ size**. Mice (n = 6) were irradiated on day 0 and then were treated with vehicle (Control), APS (50 mg/kg/day, APS) and TPO (5 ug/kg/day, TPO) as described in Figure 2. Their body weight and organ size were then measured. Effects of APS and TPO treatments on the animal weight (A), organ size (B) and organ size normalized by body weight (C) are shown. The statistical tests were carried out on the measurements of samples treated with APS and TPO and those in the control group. For samples in the control group (A), the comparisons were carried out between those of days 0, 14 and 21 and those of day 7. *: *p <*0.05; #: *p <*0.01 and +: *p <*0.001.

Animal body weights in vehicle-, APS- and TPO-treated groups were determined on days 0, 7, 14 and 21, respectively. It showed a gradually increase on days 14 and 21 (Figure 3A). However, there was a body weight decrease in vehicle- and TPO-treated animals on day 7 after irradiated treatment, but this decrease was not observed in APS treated mice (Figure 3A). It is possible that APS protects the loss of body weight resulted from irradiation. The body weights for mice treated with APS on days 14 and 21 were significantly smaller than those in the control group. Similarly, TPO treated mice showed significantly smaller body weight on days 14 (*p <*0.001) and 21 (*p <*0.001). In the comparison of organ size (Figure 3B), spleens in the APS-treated mice were significantly larger than those in the control and TPO treated groups (*p <*0.001). To determine the net effects of APS on the animal organ size, we normalized the weight of the organ to that of the body (Figure 3C). As shown, both liver (*p <*0.001) and spleen (*p <*0.001) from the APS-treated mice were significantly larger than those of the control group and the TPO treated group. The mechanisms underlying this observation remain to be elucidated.

#### APS significantly increased the formation of bone marrow CFU

**Figure 4:**
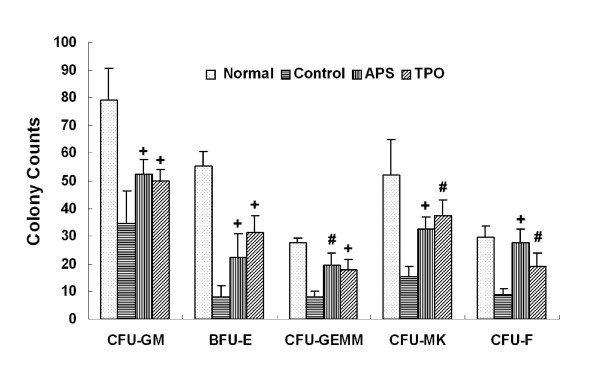
**Effects of APS on the formation of CFU of the myeloid (CFU-GM), erythroid (BFU-E), mixed (CFU-GEMM), megakaryocytic (CFU-MK) and bone marrow stromal (CFU-F) cells**. The colony counts for various CFU with or without APS treatment are shown. The statistical tests were carried out between colony counts of APS, TPO treated samples and those of control samples. Abbreviations: APS, *Angelica Sinensis *Polysaccharide; BFU/CFU-E, burst-forming unit/colony-forming unit-erythroid; CFU, colony-forming unit; CFU-E, colony-forming unit-erythroid; CFU-F, colony-forming unit-fibroblast; CFU-GM, colony-forming unit-granulocyte macrophage; CFU-GEMM: colony-forming unit-mixed; CFU-MK, colony-forming unit-megakaryocyte. #: *p <*0.01 and +: *p <*0.001.

We then tested the *in vivo *effect of APS on the formation of hematopoietic CFU of the myeloid (CFU-GM), erythroid (BFU/CFU-E), mixed (CFU-GEMM) and megakaryocytic (CFU-MK) lineages, and bone marrow stromal cells (CFU-F). Bone marrow cells in control, APS and TPO treated animals were collected and cultured for CFU assays. A pilot study has been conducted to demonstrate the dose-dependent effects of APS on bone marrow cells' productions and also to determine the optimal dose to be used in the following studies (data not shown). As shown in Figure 4 treatment with APS led to significant increase of the formation of CFU-GM, BFU-E, CFU-GEMM, CFU-MK and CFU-F. The different cell lineages, treatment groups and the corresponding mean CFU counts are shown in Table 1. Treatments that do not show significantly different effects are grouped into the same treatment groups, which are indicated by A, B, C or D respectively. As shown in Table 1 APS and TPO significantly increase the CFU counts in all cell lineages comparing to that of the control. However, APS and TPO showed similar effects for CFU-GM, CFU-GEMM and CFU-MK. For BFU-E, TPO has a much stronger effect than APS, but the effect is not strong enough to increase the CFU counts to that of the normal samples. In contrast, for CFU-F, APS has a very strong stimulating effect and it increased the CFU-F counts to the level of the normal samples (Figure 4 and Table 1).

**Table 1:** Effect of APS on the formation of colony-forming units (CFU).

Assays	Treatment Group	Mean CFU Counts	Treatment Effect Group			
CFU-GM	N	79.3	A			
	
	APS	52.3		B		
	
	TPO	50.0		B		
	
	C	34.5			C	

BFU-E	N	55.3	A			
	
	TPO	31.2		B		
	
	APS	22.3			C	
	
	C	8.3				D

CFU-GEMM	N	27.7	A			
	
	APS	19.7		B		
	
	TPO	18.0		B		
	
	C	8.3			C	

CFU-MK	N	51.8	A			
	
	APS	32.5		B		
	
	TPO	37.5		B		
	
	C	15.3			C	

CFU-F	N	29.8	A			
	
	APS	27.6	A			
	
	TPO	19.3		B		
	
	C	8.8			C	

The following CFUs, myeloid (CFU-GM), erythroid (BFU-E), mixed (CFU-GEMM), megakaryocytic (CFU-MK) and bone marrow stromal (CFU-F) have been tested for the effects of APS or TPO treatment. A, B, C and D indicates treatment groups that have significantly different treatment effects. N: Normal, cells cultured in the presence of cytokine and serum; C: Control, cells grown in cytokine serum-depleted media; APS: *Angelica Sinensis *Polysaccharide; TPO: Thrombopoietin.

#### Effects of APS on bone marrow histology

**Figure 5:**
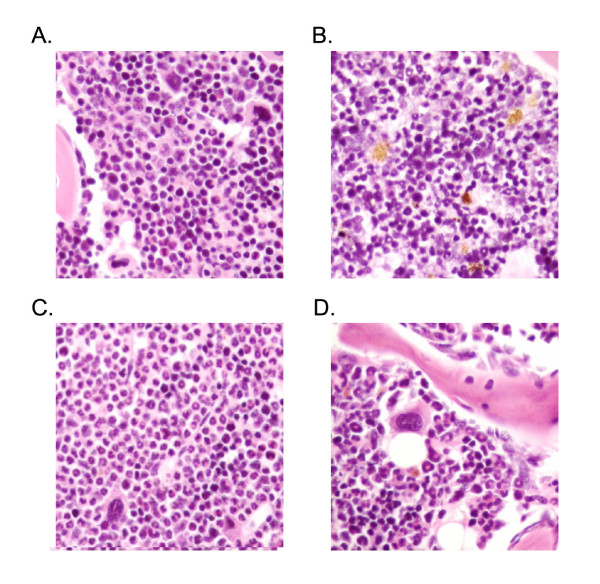
**Effects of APS on bone marrow histology**. The Wright-Giemsa stained bone marrow sections were examined for Normal (A), Control (B), APS treated (C) and TPO treated (D) mice.

We also investigated the bone marrow morphology using Wright-Giemsa staining. Twenty-five randomly selected areas of standardized size were examined (4000×) for mean total cell counts (MTC) and mean cell counts in the three cell lineages, erythroid, granulocytic and megakaryocytic series in each area. In the irradiated mice (control, Figure 5B), the hematopoiesis was markedly suppressed as evident from the dramatic drop (~50%) in the MTC/area concomitant with an increase of necrotic and apoptotic cells comparing to the normal group (Figure 5A). The reduction was particularly prominent in the granulocytic (~60%) and megakaryocytic (~100%) series but much less so in the erythroid series. In the APS treated irradiated mice, the bone marrow was hyperplastic (~ 70% increase of cellularity) comparing to the normal, which was mainly due to a prominent granulocytic expansion (Figure 5C). The megakaryocyte number was also significantly increased than that of the control, being very close to that of normal. In the TPO treated irradiated mice, trilineage hematopoiesis was preserved although the overall cellularity (MTC/area) was still slightly subnormal, ~20% reduction could be observed compared to normal (Figure 5D). The numbers of granulocytic and megakaryocytic cells were very close to that of the normal group, indicating a better recovery of these cells than the erythroid series, which showed essentially the same number as the control. In conclusion, in both the TPO and APS treated groups, there was significantly increased recovery of the megakaryocytic and granulocytic series by day 21 over the control group.

### (3) APS Exerted Antiapoptotic Effects on M-07e Cells

As shown above, APS and TPO both promoted the growth of RBCs, WBCs and platelets (Figure 2), and their corresponding progenitor cells CFU-MK, CFU-GM and BFU-E (Figure 4). Our previous studies suggest that the increase of the cell population might also result from the antiapoptotic effect of the agents in addition to the direct stimulatory effect on cell growth [14]. Consequently, we tested the antiapoptotic effect of APS using megakaryocytic cell line M-07e.

**Figure 6:**
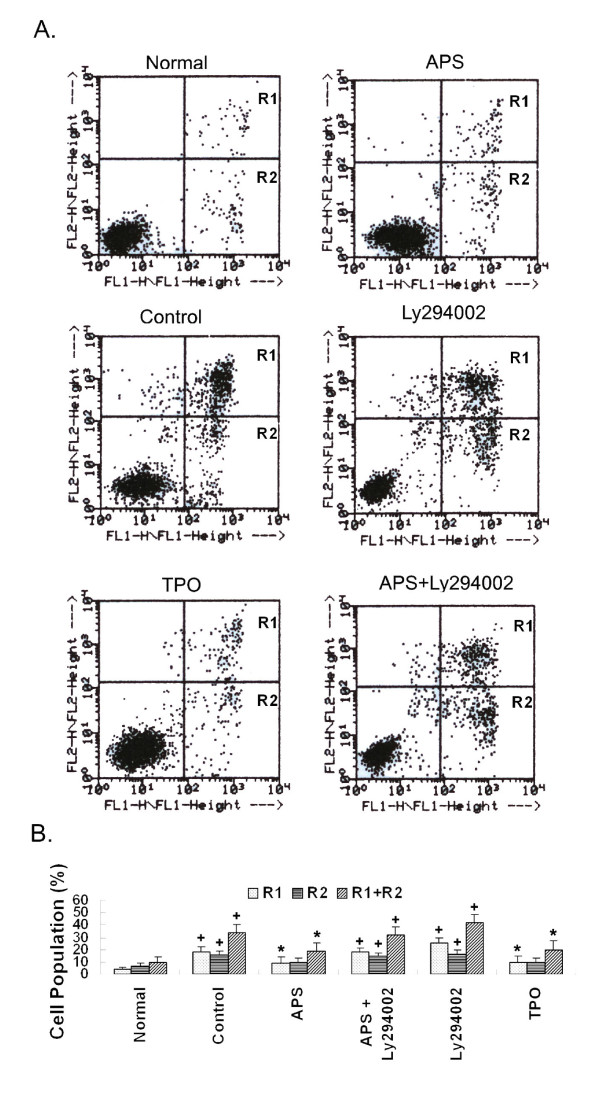
**Effects of APS on apoptosis analyzed by annexin V assay**. Apoptosis was induced by serum depletion (control). APS, TPO or Ly294002 were used to treat the cells for 72 hours. The cells were stained with annexin V-FITC and Propidium Iodide (PI) and subjected to flow cytometry analyses. (A). Dot plots of Normal, APS-treated, Control, Ly294002-treated, TPO-treated, and APS+Ly294002-treated samples. (B). Quantification and statistical analyses of treatment effects. Percentages of R1 (FITC+, PI+, late apoptotic cells), R2 (FITC+, PI-, early apoptotic cells) and R1+R2 (FITC+, total apoptotic cells) are shown. Statistical tests were carried out between the control/treated samples and the corresponding normal samples respectively. *: *p <*0.05; #: *p <*0.01 and +: *p <*0.001.

We first detected the apoptosis of cells under various treatments using the Annexin V assay (Figure 6). Annexin V protein, which is conjugated to a fluorescent dye FITC, is used to detect early and late apoptotic cells because it specifically binds Phosphatidylserine (PS), which is translocated from the inner leaflet of the plasma membrane to the outer leaflet soon after the induction of apoptosis. Another fluorescent dye Propidium Iodide (PI) is used to stain the DNA in late apoptotic and necrotic cells whose plasma membranes become permeable. The treatments are grouped based on the percentages of early (R2, FITC+ and PI-), late (R1, FITC+ and PI+) and total (R1+R2, FITC+) apoptotic cells (Table 2). As shown, the lowest percentage of apoptotic cells were seen in the normal samples and the highest percentage of apoptotic cells were seen in the Ly294002 treated samples, while intermediate percentages of apoptotic cells were seen in the control samples. Treatments with APS and TPO significantly reduced the early (R2), late (R1) and total (R1+R2) apoptotic cells comparing to those of the control and their effects are not significantly different. Comparing to treatment with Ly294002 alone, additional APS treatment reduced the percentages of apoptotic cells from 25.75% to 17.76% for late apoptotic cells (R1) and from 41.88% to 32.25% in total apoptotic (R1+R2) cells. Interestingly, no differences in percentages of early apoptotic cells were observed among the Ly294002-treated (16.13%), Control (15.71%) and APS+L-treated (14.49%) samples, suggesting that APS might function mainly in preventing cells from proceeding to the late apoptotic phase.

**Table 2:** Effects of various treatments on the apoptosis of M-07e cells detected by annexin V assay.

	Treatment Group	Percentage of Cells (%)	Treatment Effect Group			
R1	L	25.75	A			
	
	APS + L	17.76		B		
	
	C	17.75		B		
	
	TPO	9.87			C	
	
	APS	9.21			C	
	
	N	3.86				D

R2	L	16.13	A			
	
	C	15.71	A			
	
	APS +L	14.49	A			
	
	APS	9.65		B		
	
	TPO	9.60		B		
	
	N	6.19		B		

R1+R2	L	41.88	A			
	
	C	33.47		B		
	
	APS + L	32.25		B		
	
	TPO	19.47			C	
	
	APS	18.87			C	
	
	N	10.05				D

A, B, C and D stand for treatment groups having significantly different treatment effects. R1: late apoptotic cells; R2: early apoptotic cells; R1+R2: total apoptotic cells; N: Normal, cells cultured in the presence of cytokine and serum; C: Control, cells grown in cytokine and serum-depleted media; APS: *Angelica Sinensis *Polysaccharide; TPO: Thrombopoietin. L: Ly294002.

**Figure 7:**
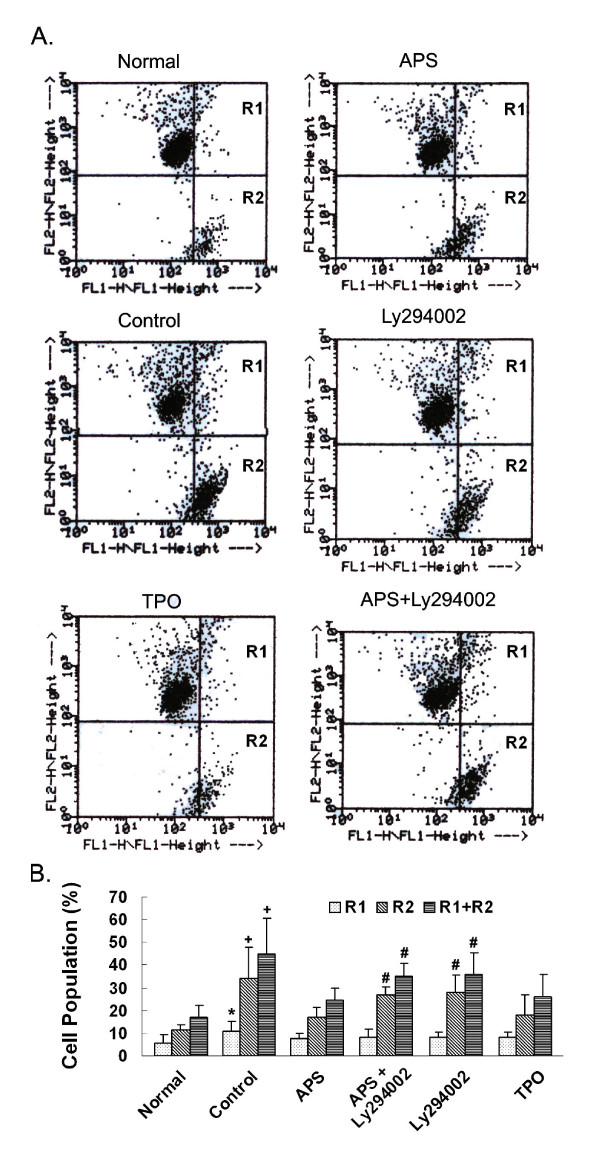
**Effects of APS on apoptosis analyzed by JC1 assay**. M-07e cells were treated as described above, incubated with JC-1 reagent and then subjected to flow cytometry analyses. (A). Dot plots of Normal, APS-treated, Control, Ly294002-treated, TPO-treated, and APS+Ly294002-treated samples. (B). Quantification and statistical analyses of treatment effects. Percentages of R1 (transitional cell subset containing both JC1 aggregates and monomers, representing early apoptotic cells), R2 (apoptotic cells containing mostly monomers, representing late apoptotic cells), R1+R2 (total apoptotic cells) cells are shown. Statistical tests were carried out between the control/treated samples and the corresponding normal samples respectively.*: *p <*0.05; #: *p <*0.01 and +: *p <*0.001.

Then, we confirmed the anti-apoptosis effects of APS by JC-1 assay. JC-1 is one type of compound that aggregate in mitochondria in normal cells (Figure 7A, red fluorescence, detected in FL2) giving off red fluorescence. In apoptotic cells, the mitochondria transmembrane potential (m) breaks down, causing JC-1 to remain in the cytoplasm in its monomer form and fluorescing green (Figure 7A, green fluorescence, detected in FL1). We measured the apoptotic cell populations based on the distribution of JC-1 in aggregate and monomer forms (Figure 7 and Table 3). R1 represents cells contain both JC1 aggregates and monomers, which are early apoptotic cells. R2 represents cells contain only JC1 monomers, which are late apoptotic cells. R1+R2 thus represent all apoptotic cells (Figure 7A). Compared with normal M-07e cells, cells from control, Ly294002-treated, APS+Ly294002-treated samples had significantly increased proportions of cells containing JC-1 monomers (Figure 7B and Table 3), indicating the induction of apoptosis in these samples comparing to normal samples. Interestingly, no significant differences were observed between apoptotic cells in APS or TPO treated samples comparing to normal samples, indicating the protective effects of APS and TPO treatments.

**Table 3:** Effects of various treatments on the apoptosis of M-07e cells detected by JC1 assay.

	Treatment Group	Percentage of Total Cell Population (%)	Treatment Effect Group		
R1	C	11.08	A		
	
	TPO	8.10	A	B	
	
	L	8.04	A	B	
	
	APS + L	7.83	A	B	
	
	APS	7.61	A	B	
	
	N	5.75		B	

R2	C	33.84	A		
	
	L	28.01	A		
	
	APS + L	26.95	A	B	
	
	TPO	17.84		B	C
	
	APS	17.06		B	C
	
	N	11.42			C

R1+R2	C	44.91	A		
	
	L	36.05	A	B	
	
	APS + L	34.78	A	B	
	
	TPO	25.94		B	C
	
	APS	24.68		B	C
	
	N	17.17			C

A, B, C and D stand for treatment groups having significantly different treatment effects. N: Normal, cells cultured in the presence of cytokine and serum; C: Control, cells grown in cytokine and serum-depleted media; R1: early apoptotic cell; R2: late apoptotic cell; R1+R2: total apoptotic cell; APS: *Angelica Sinensis *Polysaccharide; TPO: Thrombopoietin. L: Ly294002.

**Figure 8:**
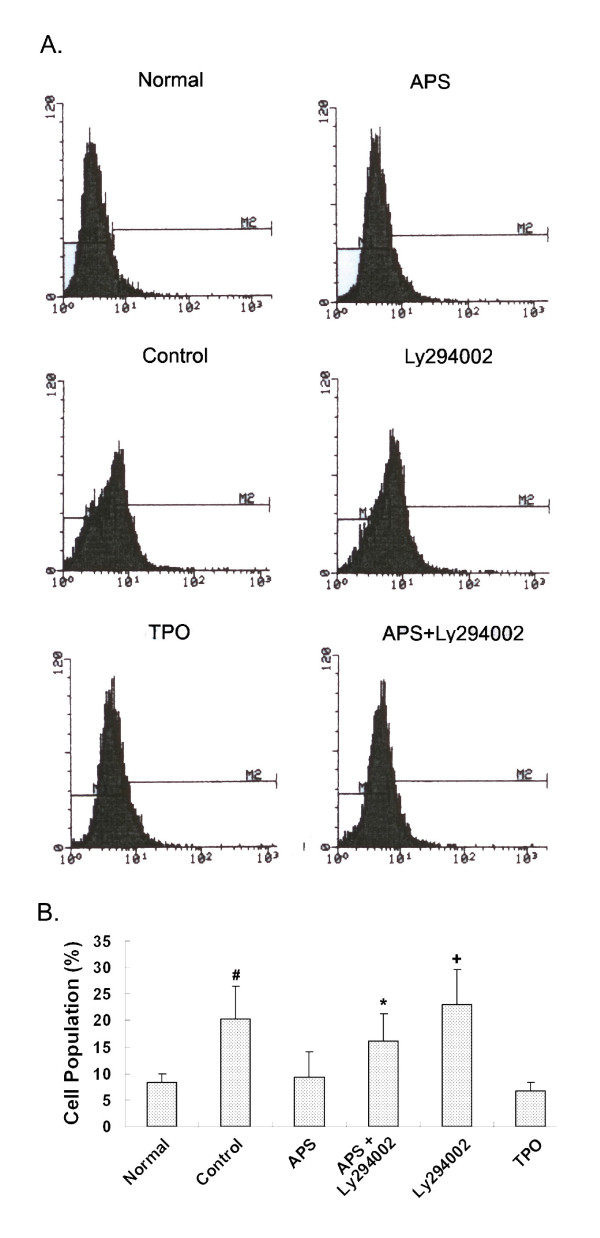
**Effects of APS on apoptosis analyzed by Caspase 3 assay**. M-07e cells were treated as described above, stained with Caspase 3-PE (FL-2) and subjected to flow cytometry analysis. (A). Histogram of Normal, APS-treated, Control, Ly294002-treated, TPO-treated and APS+Ly294002-treated samples. (B). Quantification and statistical analyses of treatment effects. Statistical tests were carried out between the control/treated samples and the corresponding normal samples respectively. *: *p <*0.05; #: *p <*0.01 and +: *p <*0.001.

Last, we analyzed the anti-apoptosis effects of APS using the Caspase 3 assay. Caspase 3 is a downstream effector protein of apoptosis, expression of which is indicative of undergoing apoptosis. M-07e cells were treated as described, labelled with Caspase 3-PE dye and then subjected to flow cytometry analysis. Histograms of samples under various treatments are shown in Figure 8A. Statistical testing between the indicated samples and the normal samples are shown in Figure 8B. More detailed statistical testing results are summarized in Table 4 in the same manner as those for Annexin V assay and JC-1 assay. As shown in Figure 8B and Table 4 percentages of cells expressing Caspase 3 in APS-treated, TPO-treated samples are similar to those of the normal. Ly294002-treated and the control samples had significantly higher percentages of cells expressing Caspase 3 activity, indicative of these treated cells have undergone apoptosis. APS+Ly294002 treatment significantly decreased the percentage of cells expressing Caspase 3 activity to 16.02% comparing to that of Ly294002 alone (23.15%).

**Table 4:** Effects of various treatments on the apoptosis of M-07e cells detected by Caspase 3 assay.

Treatment Group	Percentage of Total Cell Population (%)	Treatment Effect Group			
L	23.15	A			

C	20.38	A	B		

APS + L	16.02		B	C	

APS	9.41			C	D

N	8.40				D

TPO	6.83				D

A, B, C and D stand for treatment groups having significantly different treatment effects. N: Normal, cells cultured in the presence of cytokine and serum; C: Control, cells grown in cytokine and serum-depleted media; APS: *Angelica Sinensis *Polysaccharide; TPO: Thrombopoietin. L: Ly294002.

## Discussion

In our previous study, we showed that DBT extracts can promote hematopoiesis and thrombopoiesis [4]. Since APS represents a significant portion of DBT extracts, we hypothesize that APS may have effects similar to that of DBT. As we demonstrated in our experiments using mouse and cell models, APS showed hematopoietic and thrombopoietic effects similar to those of DBT. These led us to conclude that APS represents the major active components in DBT. Two questions have been raised here. The first one is what the active component of APS is. APS contains lipid component, it is conceivable that the plant-derived lipopolysaccharide (LPS) is the major active component. Since we did detect low level of endotoxin activity in APS samples using the TAL test, we cannot exclude the participation of endotoxin-like activity in our experiments. However, what percentage of the total activities we detected in this study can be attributed to this TAL detected activity remains to be determined.

The second question is what the molecular targets of APS are. Previous studies suggest that the cellular recognition of LPS is mainly mediated through the interaction with three proteins, TLR4, CD14, and MD2. LPS binds to the serum protein LPS-binding protein, and the resulted complex can be recognized by CD14, a cell-surface 55-kDa glycoprotein with a glycosyl-phosphatidyl-inositol membrane anchor [18]. MD2 is a 20-30 kDa glycoprotein that binds to the extracellular domain of TLR4 [19], which was identified as an LPS receptor [20,21]. Several recent studies have further implicated the three proteins in LPS-induced cellular response. For example, LPS can induce physical proximity between CD14 and TLR4 [22]; LPS is in close proximity to TLR4 in the presence of CD14 and MD2 [23] and TLR4 and MD2 provide greater specificity for ligands and more efficient responsiveness to LPS [19].

TLR4 is a member of the Toll-like receptors (TLRs) family, which play a critical role in inducing innate immune responses in mammals by recognizing conserved pathogen-associated molecular patterns of bacteria [24-26]. So far, 10 human TLRs have been cloned [27-30]. The TLR agonists include lipopolysaccharide (LPS) for TLR4, peptidoglycan for TLR2 and TLR6, double-stranded RNA for TLR3, flagellin for TLR5, and imidazoquinolines and unmethylated CpG motifs in bacterial DNA for TLR7 and TLR9, respectively [31-34]. TLR4 can be activated by nonbacterial agonists such as HSP60, fibronectin, Taxol, respiratory syncytical virus coat protein, and saturated fatty acids [35-40].

In terms of the downstream signalling pathway, the PI3K/AKT Pathways has been suggested as one of the downstream signalling pathways of TLRs. Wortmannin, a PI3K inhibitor, inhibits NF B activation and cytokine production by CpG DNA, a TLR9 agonist and LPS [41,42]. B lymphocytes deficient in PI3K regulatory units failed to respond to LPS [43]. The stimulation of TLR2 by *Staphylococcus aureus *induces the recruitment of PI3K and the activation of AKT, leading to NF B transactivation [44]. These results demonstrate the role of PI3K in TLR signalling pathways in general. However, TLR4 does not have a PI3K-binding site. A recent model suggests that PI3K/AKT is the downstream signalling component of the MyD88-dependent TLR4 signalling pathway in particular [45]. Future experiments are needed to test this MyD88-dependent hypothesis in details. The results obtained from this study also set the stage for addressing other important questions, such as the specificities of APS, if there are any; and whether or not other chemical components of DBT have similar or complementary effects.

## Conclusions

Here, we showed that APS, the polysaccharide-enriched extracts from the root of *Angelica sinensis *has hematopoietic and thrombopoietic activities *in vivo *in a mouse model. In M-07e cells, we showed that APS can protect cells from undergoing apoptosis and its effects involved the PI3K/AKT Pathways. Taking together, APS's hematopoietic and thrombopoietic effects are likely to result from the activation of the PI3K/AKT Pathways, the activation of which protects cells from undergoing apoptosis.

## Competing interests

The authors declare that they have no competing interests.

## Authors' contributions

CL participated in the design of the study, performed the statistical analyses of all data and drafted the manuscript. JQL and HXY carried out the endotoxic assay. RXD carried out the chemical characterization experiments. MY conceived the study. MY, NHP, CPL, SWC, JLC and STY carried out the animal and cellular studies. JYY carried out the Apoptosis assays. SXL, FYM, CKL, SLC and BHC critically reviewed the manuscript. All authors have read and approved the final manuscript.

## Pre-publication history

The pre-publication history for this paper can be accessed here:

http://www.biomedcentral.com/1472-6882/10/79/prepub
